# Prevalence and association of single nucleotide polymorphisms with sarcopenia in older women depends on definition

**DOI:** 10.1038/s41598-020-59722-9

**Published:** 2020-02-19

**Authors:** Praval Khanal, Lingxiao He, Georgina Stebbings, Gladys L. Onambele-Pearson, Hans Degens, Alun Williams, Martine Thomis, Christopher I. Morse

**Affiliations:** 1 Department of Sport and Exercise Sciences, Musculoskeletal Science and Sports Medicine Research Centre, Manchester Metropolitan University, Manchester, UK; 2 Department of Movement Sciences, Physical Activity, Sports & Health Research Group, KU Leuven, Leuven, Belgium; 3 Department of Life Sciences, Manchester Metropolitan University, Manchester, UK; 4 Institute of Sport Science and Innovations, Lithuanian Sports University, Kaunas, Lithuania; 5 University of Medicine and Pharmacy of Targu Mures, Targu Mures, Rumania; 6 Institute of Sport, Exercise and Health, University College London, London, UK

**Keywords:** Genetic association study, Genotype

## Abstract

The prevalence of sarcopenia depends on the definition used. There are, however, consistent sarcopenic characteristics, including a low muscle mass and muscle strength. Few studies have investigated the relationship between sarcopenia and genotype. A cross-sectional study was conducted with 307 community-dwelling ≥60-year-old women in South Cheshire, UK. Handgrip strength was assessed with a handgrip dynamometer and skeletal muscle mass was estimated using bioelectrical impedance. DNA was extracted from saliva (∼38%) or blood (∼62%) and 24 single-nucleotide polymorphisms (SNPs) were genotyped. Three established sarcopenia definitions - %Skeletal Muscle Mass (%SMM), Skeletal Muscle Mass Index (SMI) and European Working Group on Sarcopenia in Older People (EWGSOP) - were used to assess sarcopenia prevalence. Binary logistic regression with age as covariate was used to identify SNPs associated with sarcopenia. The prevalence of sarcopenia was: %SMM 14.7%, SMI 60.6% and EWGSOP 1.3%. Four SNPs were associated with the %SMM and SMI definitions of sarcopenia; *FTO* rs9939609, *ESR1* rs4870044, *NOS3* rs1799983 and *TRHR* rs7832552. The first three were associated with the %SMM definition, and *TRHR* rs7832552 with the SMI definition, but none were common to both sarcopenia definitions. The gene variants associated with sarcopenia may help proper counselling and interventions to prevent individuals from developing sarcopenia.

## Introduction

Sarcopenia is defined as an ageing-related loss of both muscle mass and strength below a threshold level^1^. It is an important predictor of adverse outcomes such as limited mobility, increased risk of falls, decreased quality of life (QoL), hospitalization and mortality, and contributes to tens of millions of pounds of health care costs in the UK^1–3^. Although muscle weakness and skeletal muscle atrophy are overt characteristics of this geriatric syndrome, there is ongoing debate on the operational definition, screening and diagnosis, and optimal management and treatment of the condition^1,4–6^. The considerable heterogeneity in the reported prevalence of sarcopenia is largely attributable to the different definitions or cut-offs used^7–9^.

The fact that some elderly do not show sarcopenia, whilst others of the same age do^10,11^, suggests that some individuals are more susceptible to sarcopenia than others. The different susceptibility is likely due to a combination of factors including physical activity, diet, sedentary behaviour and genetics^12–15^. Several studies have reported an association of single nucleotide polymorphisms (SNPs) with lean mass, muscle volume and muscle strength^16–18^. It is thus possible that individuals carrying favourable gene variants are less susceptible to sarcopenia and hence can maintain independence until later life. To date, five studies have investigated the association of SNPs with sarcopenia; limited to *VDR*, *IL6*, *ACTN3* and *MSTN* polymorphisms^19–23^. The studies identified an association of *ACTN*3 and *VDR* gene variants with sarcopenia, but did not find any association with *IL6* and *MSTN* variants. Unlike the previous studies that used low appendicular skeletal muscle mass as the cut-off for sarcopenia^19,20,22^, the later studies used both low muscle mass and muscle function to define sarcopenia^21,23^.

The genetic (as opposed to environmental) component of inter-individual differences in muscle size/strength are substantial, both in younger^24,25^ and older adults^26^. To date however, research in older adults has attempted to identify the precise genetic variations responsible for sarcopenia through four candidate SNPs, and none has compared different sarcopenia definitions in that regard. It would be advantageous to do so, with desirable outcomes including a greater understanding of the differences observed and the possibility of targeting interventions at those most at risk of sarcopenia. Considering the possible association of gene variants with sarcopenia (although the results may vary with different sarcopenia definitions) and heterogeneity in sarcopenia prevalence, the objectives of the present study were to assess 1) the prevalence of sarcopenia in a population of older women according to three different definitions of sarcopenia and 2) the association between sarcopenia and more SNPs than studied previously, for each definition of sarcopenia. The three chosen definitions were: skeletal muscle mass (SMM)/body mass (%SMM) (sarcopenia defined to individuals with %SMM < 22.1%)^27^, SMM/height^2^ (SMI) (sarcopenia defined to individuals with SMI ≤ 6.76 kg/m^2^)^28^ and SMI and Handgrip strength (HGS) cut-offs as suggested by European Working Group on Sarcopenia in Older People (EWGSOP) (sarcopenia defined to individuals with SMI < 6.76 kg/m^2^ and HGS < 20 kg)^1^.

## Results

### Prevalence of sarcopenia with different sarcopenia definitions

The prevalence of sarcopenia according to each definition was: %SMM 14.7%, SMI 60.6% and EWGSOP 1.3%. The general characteristics of the participants with each definition are presented in Table 1. Individuals identified as sarcopenic with EWGSOP were also sarcopenic by the SMI definition, while there was a discrepancy in individuals identified as sarcopenic between the SMI and %SMM definitions: 34 elderly women were sarcopenic using both the %SMM and SMI definitions, but 11 were sarcopenic using only the %SMM definition and 152 were sarcopenic using only the SMI definition. Similarly, two individuals were common to both %SMM and EWGSOP definitions, but 2 were with the EWGSOP only and 43 using only the %SMM definition.

**Table 1:** Population and sarcopenia group characteristics with different sarcopenia definition.

Sarcopenia definitions (in columns) |Variables (in rows)	General characteristics (n = 307)	SMI		%SMM		EWGSOP	
		S 186 (60.6%)	NS	S 45 (14.7%)	NS	S 4 (1.3%)	NS
Age (years)	70.7 ± 5.7	71.0 ± 5.3	70.3 ± 6.3	71.6 ± 5.6	70.6 ± 5.7	76.8 ± 7.0*	70.6 ± 5.7
Body Mass (kg)	66.3 ± 11.3	63.4 ± 9.3**	70.9 ± 12.5	77.4 ± 13.1**	64.4 ± 9.8	68.5 ± 10.9	66.3 ± 11.3
BMI (kg/m^2^)	25.9 ± 4.2	24.7 ± 3.3**	27.8 ± 4.7	30.2 ± 5.5**	25.2 ± 3.4	28.2 ± 2.9	25.9 ± 4.2
HGS (kg)	29.9 ± 5.0	29.1 ± 4.4**	31.1 ± 5.6	28.4 ± 4.9*	30.2 ± 5.0	17.8 ± 2.2**	30.0 ± 4.9
SMI (kg/m^2^)	6.56 ± 0.81	6.05 ± 0.51**	7.33 ± 0.53	6.04 ± 0.92**	6.64 ± 0.75	6.29 ± 0.18	6.56 ± 0.81

*And ** denote difference from non-sarcopenia group at p < 0.05 and p < 0.001 respectively. BMI, Body Mass Index, HGS, Hand Grip Strength, SMI, Skeletal Muscle Mass Index, S- Sarcopenia group, NS- Non sarcopenia group, n (%), number (percentage) of sarcopenic individuals.

### Associations of SNPs with sarcopenia according to the different definitions

**Figure 1:**
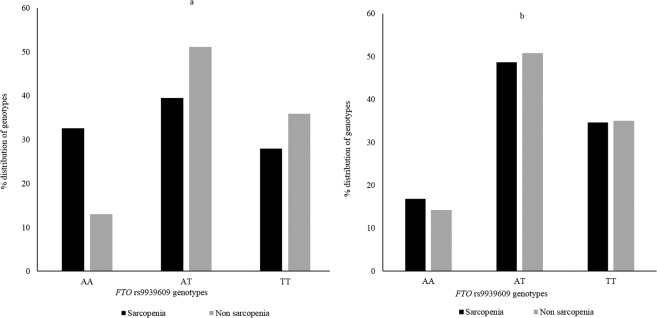
Distribution of *FTO* rs9939609 genotypes between sarcopenia and non-sarcopenia groups with sarcopenia definitions (**a**) %SMM and (**b**) SMI. Using %SMM, AA homozygotes had over three times higher risk of being sarcopenic than T-allele carriers (OR = 3.04, p = 0.004).

All genotypes were in Hardy Weinberg equilibrium (p > 0.05) (presented in Supplementary Table S1). The distribution of genotypes varies with the sarcopenia definitions (presented for *FTO* rs9939609 in Fig. 1).

Based on the %SMM definition, the SNPs *FTO* rs9939609, *ESR1* rs4870044 and *NOS3* rs1799983 were associated with sarcopenia (presented in Table 2). Binary logistic regression, using age as a covariate, indicated that *FTO* rs9939609 AA homozygotes had 3.04 times higher risk of being sarcopenic than T-allele carriers (OR = 3.037, 95% confidence interval [CI] = 1.439–6.411, p = 0.004). *ESR1* rs4870044 T-allele carriers had a 2.54 times greater risk of being sarcopenic than CC homozygotes (OR = 2.543, 95% CI = 1.273–5.079, p = 0.008). Similarly, *NOS3* rs1799983 GG homozygotes had 2.26 times greater risk of being sarcopenic than T-allele carriers (OR = 2.257, 95% CI = 1.165–4.373, p = 0.016).

**Table 2:** Binary logistic regression model of single nucleotide polymorphisms with age and PASE on sarcopenia.

Sarcopenia definitions	Variables	Β	S.E (β)	Wald's χ2	p	OR	95% CI	Risk genotypes
SMI	Age	−0.260	0.021	1.463	0.226	0.975	0.935–1.016	CC+CT
	*TRHR* rs7832552	0.943	0.360	6.858	0.009*	2.568	1.268–5.203	
	PASE	<0.001	0.002	0.005	0.944	1.000	0.996–1.005	
%SMM	Age	−0.032	0.029	1.268	0.260	0.968	0.915–1.024	AA
	*FTO* rs9939609	1.111	0.381	8.492	0.004*	3.037	1.439–6.411	
	PASE	0.008	0.004	3.735	0.053	1.008	1.000–1.015	
	Age	−0.031	0.028	1.222	0.269	0.970	0.919–1.024	GG
	*NOS3* rs1799983	0.814	0.337	5.820	0.016*	2.257	1.165–4.373	
	PASE	0.009	0.004	4.814	0.028	1.009	1.001–1.016	
	Age	−0.031	0.028	1.308	0.253	0.969	0.918–1.023	TT+CT
	*ESR1* rs4870044	0.933	0.353	6.988	0.008*	2.543	1.273–5.079	
	PASE	0.009	0.004	4.787	0.029*	1.009	1.001–1.016	

*Denotes association of SNPs and sarcopenia in a model.

Based on the SMI definition, elderly females with the *TRHR* rs7832552 C-allele carriers had 2.6-fold higher risk of being sarcopenic when compared with TT homozygotes (OR = 2.568, 95% CI = 1.268–5.203, p = 0.009).

Using the EWGSOP definition, only four participants were defined as sarcopenic, therefore comparisons of genetic characteristics between sarcopenic and non-sarcopenic groups could not be performed.

None of the other 20 SNPs were associated with sarcopenia using any definition.

## Discussion

The current study identified that different definitions of sarcopenia result in a widely different prevalence of sarcopenia, ranging from 1.3% to 60.6% in the present population of women aged >60 years. Three SNPs were associated with sarcopenia based on the %SMM definition, and one SNP when using the SMI definition.

The heterogeneity of the prevalence of sarcopenia (1.3% to 60.6%), depending on sarcopenia definition used, is consistent with a previous study^8^. This confirms that the criteria, definition and threshold has a dramatic impact on the observed prevalence of sarcopenia, even in the same study population. Defining sarcopenia using %SMM resulted in a prevalence of 14.7% in the present elderly female population. This prevalence falls within the range in previous studies using the same approach. For example, in the large population NHANES III study 10% of US women above 60 years^27^ and 23.6% of elderly French women^29^ were sarcopenic according to this definition. The SMI definition of sarcopenia has previously resulted in prevalence ranging from 2.8%–42.0%^29–31^. At the extremes, this lower prevalence in sarcopenia is due to threshold levels being set arguably too conservatively (SMI 6.2 kg/m^2^ previously)^29^, compared to 6.76 kg/m^2^ (SMI) in the present study definition consistent with previous work^28^. The prevalence of sarcopenia (1.3%) in elderly females in the present study using the EWGSOP definition is consistent with studies on different populations - for instance, 2.5% in Taiwanese^32^, 4.5% in German^33^ and 6.8% in Japanese^34^ women. It should be noted that higher prevalences have been reported, ranging between 22-48% in some of the studies conducted across Europe, Asia and South America^35–39^, but these higher prevalances tend to be in populations incorporating the oldest old (>70 years) or those not living independently and/or their own young reference group (rather than cut offs).

Within this study, elderly women identified as sarcopenic using the EWGSOP definition were also sarcopenic using the SMI definition. The SMI definition used ≤6.76 kg/m^2^ as the cut-off for low muscle mass in defining sarcopenia, while EWGSOP identified the sarcopenic with the same cut-off for low muscle mass and additionally <20 kg handgrip strength that evidently made no material difference in our cohort. However, the discrepancy in those identified as sarcopenic between those two definitions and the %SMM definition could be because the %SMM definition uses only values of mass while the other two both adjust values of mass according to height. The difference in the prevalence of sarcopenia by considering height and body mass as normalizing factors has been observed in a previous study^40^. These observations clearly indicate that a consensus is needed concerning the definition of sarcopenia.

The present study identified *FTO* rs9939609 AA homozygotes to be at over 3-fold higher risk for sarcopenia compared to T-allele carriers. This genotype has been previously associated as a risk genotype for obesity-related indices such as increased BMI and fat mass^41–43^, and clinical conditions such as type 2 diabetes^44^ and tuberculosis^45^. The importance of FTO during skeletal muscle development and differentiation was reflected by the impaired skeletal muscle development in FTO-deficient mice, due to myogenic suppression^46^. There was an association between *FTO* rs9939609 AA and lean body mass; however, that association disappeared when controlling for fat mass^41,47^. Recent studies have reported over-representation of *FTO* rs9939609 AA genotypes in heavier athletes^48,49^, however, none has investigated how the body mass and muscle mass are affected by this genotype in those athletic populations. We previously demonstrated that the T-allele is associated with higher appendicular and arm lean mass in untrained young men^49^. It therefore appears that *FTO* AA-genotype individuals have lower skeletal muscle mass and are at higher risk of sarcopenia.

The current study is the first to identify the *NOS3* rs1799983 GG genotype as a risk factor for sarcopenia, with over 2-fold higher risk compared to T-allele carriers, for sarcopenia in the elderly. This finding is consistent with previous studies that have reported the T allele as more frequent in power oriented athletes^50^ and female short distance swimmers^51^ and association of TT homozygote with absolute gain in dynamic strength following resistance training in women^52^. Biologically, the *NOS3* gene encodes the enzyme endothelial nitric oxide synthase (eNOS) that catalyses the synthesis of nitric oxide (NO). NO has been identified as a determinant of individual variations in health and exercise related phenotypes^53^, mitochondrial energy production^54^ and muscle hypertrophy^55^. The *NOS3* rs1799983 T allele has been associated with higher NOS activity compared to the G-allele^56^. The association of the *NOS3* rs1799983 T allele with a higher NOS activity and the beneficial role of NO in skeletal muscle, particularly muscle hypertrophy and energy production, provide a potential basis for the present observation that elderly women who are GG homozygotes are at higher risk of being sarcopenic.

The current study also identified the *ESR1* rs4870044 T allele as a risk factor, with 2.5-fold higher risk of sarcopenia in T-allele carriers compared to CC homozygotes. Biologically, *ESR1* encodes the oestrogen receptor protein that interacts with oestrogen and plays an important role in growth of bone and maintenance of bone mass. ESR1 is also expressed in human skeletal muscle^57^. However, the role of oestrogen in skeletal muscle is still to be elucidated. The *ESR1* rs4870044 T allele has been associated previously with low bone mineral density (BMD)^58,59^ and a higher risk of hip fracture^60^. Since ageing results in the deterioration of both bone and muscle, and bone geometry is partly determined by muscle mass/strength^61,62^, *ESR1* might contribute to both muscle and bone phenotypes^63^. Although previous studies did not find an association between rs4870044 and muscle strength^64–66^, we suggest that the discrepancy between those studies and our study might be attributable to us defining sarcopenia by the %SMM while the other studies assessed the relationship with muscle strength. Whatever the cause of the discrepancy, the higher risk of sarcopenia in T-allele carriers may be related to the physiological activities of oestrogen, mediated by oestrogen receptors^67,68^ where lower levels of oestrogen have previously been associated with low bone mass and bone mineral density^69,70^, as well as low muscle mass^71^ and muscle strength^71,72^.

In the current study, *TRHR* rs7832552 C-allele carriers were at over 2-fold higher risk compared to TT homozygotes for sarcopenia defined according to the SMI. This aligns with studies associating the T-allele with increased muscle mass. For example, a genome-wide association study identified individuals homozygous for the *TRHR* rs7832552 T-allele to have on average 2.5 kg more lean body mass than heterozygotes and C-allele homozygotes^73^. Similarly, this polymorphism has been associated with sprint/power performance^74^. A greater sarcopenic risk for C-allele carriers in the present elderly might be explained by lower *TRHR* expression^75^ and hence impaired action of thyroid hormone that is considered important to preserve muscle strength^76^.

The prevalence and associations of sarcopenia and SNPs were studied with three different sarcopenia definitions. One limitation is that the current study was limited to older women only, but then particularly women suffer from (the consequence of) sarcopenia. Another limitation is that SMM was estimated using BIA instead of the more accurate MRI or DEXA. Although many studies have shown a good agreement (e.g. standard error of estimate 2.7 kg^77^) between BIA and MRI/DEXA^77–79^, BIA might overestimate muscle mass^80,81^. Therefore, future studies that adopt a more rigorous approach to assessing muscle mass (MRI or DXA) may observe stronger SNP associations than those reported in the present study. As diet^82^ also plays an important role in the maintenance of muscle mass/strength, we also encourage future work that incorporates diet and those additional possible confounders to understand the sarcopenia mechanism in depth.

Within our study, only four of the twenty-four investigated SNPs were associated with sarcopenia. Skeletal muscle mass and strength are highly polygenic in nature^83^, hence the influence of any single SNP on the risk of sarcopenia is probably limited. A polygenic approach combining multiple risk alleles for sarcopenia will ultimately capture a substantial portion of the genetic risk and any future practical tool will use that approach. However, many more individual SNPs first need to be identified before a polygenic approach becomes a worthwhile development. It should also be noted that the sarcopenia definitions identified different SNPs to be associated with sarcopenia. This is probably because different individuals were identified as sarcopenic by the different definitions and demonstrates how inconsistency in the definition of sarcopenia in the literature will have consequences for the ability to identify causative factors. Given the perpetual decline of muscle mass and strength in the elderly, although individuals in our study identified as sarcopenic using the SMI and %SMM definitions (in fact they were independent and could perform daily activities without any limitations), they are probably at a higher risk of developing sarcopenia and doing so earlier than individuals not identified as sarcopenic using any definition. As a genetic association was found, SNP- related risk seems pertinent even in those elderly who are currently healthy. Similarly, the tested SNPs were not selected randomly, but due to their previous association with skeletal muscle phenotypes or similar phenotypes, so we believe the results observed are informative regarding sarcopenia-related phenotypes.

The prevalence of sarcopenia is to a large extent affected by the definition used, and ranged in our population of older women from 1.3 to 60.6%. This clearly shows that, when comparing studies, due attention must be given to the definition of sarcopenia. We also identified four SNPs (*FTO* rs9939609, *ESR1* rs4870044, *NOS3* rs1799983 and *TRHR* rs7832552) associated with sarcopenia and this information might be used (with other data) to identify individuals at a higher risk of sarcopenia and facilitate early targeted intervention to offset that higher risk.

## Methods

### Participants

Participants comprised 60- to 91-year-old Caucasian women (n = 307; 70.7 ± 5.7 years, 66.3 ± 11.2 kg, 1.60 ± 0.06 m; mean ± SD). All participants were recruited from social groups, and subsequent word-of-mouth between participants. These social groups ensured the participants were either physically active at a recreational level (e.g. table tennis, walking groups, dance classes) and societally engaged (e.g. book groups, and discussion groups). All were independently living, ambulatory and had no history of severe muscle and bone issues such as osteoporosis or rheumatoid arthritis, nor cancer, cardiovascular-related diseases or nervous system disorders such as Alzheimer’s, convulsions or epilepsy.

### Skeletal muscle mass index

Skeletal muscle mass was quantified using Bioelectrical Impedance Analysis (BIA) (Model 1500; Bodystat, Isle of Man, UK). Although Dual-Energy X-ray Absorptiometry (DEXA) is commonly used for measuring appendicular lean mass index (ALMI) in sarcopenia studies^8,84^, BIA has been suggested as a valid and low-cost alternative for measuring Skeletal Muscle Mass Index (SMI) and is a common tool in larger population studies^27,85^. Indeed, a high correlation between muscle mass measured with BIA and DEXA has been reported^78,79^. Participants were instructed to lie on a physiotherapist bed in a supine position with both upper and lower limbs slightly abducted from the body for about 4–5 min. Two adhesive electrodes were placed on the dorsum of the hand and foot on the right side of the body. An electrical current (frequency: 50 kHz; amplitude: 0.4 mA) was then passed between these electrodes and the skeletal muscle mass was estimated using an established formula^77^:

$${\rm{Skeletal}}\,{\rm{Muscle}}\,{\rm{Mass}}\,({\rm{SMM}})=[{{\rm{Ht}}}^{2}/({\rm{R}}\,\times \,0.401)+({\rm{sex}}\,\times \,3.825)+({\rm{age}}\,\times -0.071)]+5.102$$

Where Ht is height in cm, R is resistance in Ω and age in years. For sex, a male is scored as 1 and female as 0. SMI as SMM/Ht^2^ was calculated using SMM in kg and height of the participant in m.

### Handgrip strength

Handgrip strength (HGS) was measured using a digital load cell handgrip dynamometer (JAMAR plus, JLW Instruments, Chicago, USA) with a previously validated protocol^86^. Participants were instructed to stand in an upright position with the dynamometer held with the arm straight, and flexed at 90° to the shoulder. Verbal encouragement was provided to each participant to squeeze the handgrip dynamometer with maximum force, which was maintained for 5 s. The left and right arm were alternated, with 1 min between trials. The highest grip strength of three maximal efforts on each arm was recorded. The test-retest reliability of HGS in the dominant hand is high (ICC = 0.99) in healthy elderly participants^87^.

### Physical activity scale for elderly questionnaire

Participants completed the Physical Activity Scale for Elderly (PASE) questionnaire in the lab on the testing day. The questionnaire is a 7-day recall questionnaire identifying time spent undertaking activities such as sitting, moderate intensity activities, recreational activities, strenuous activities and endurance and muscle strength related exercises. The questionnaire includes questions related to time spent in household work, gardening, caring for a dependent person, and work (paid or voluntary). The total PASE score was computed by multiplying the amount of time spent in each activity (hours/week) or participation (yes/no) in an activity by the empirically derived item weights and summing over all activities^88^.

### DNA sample collection, DNA extraction, SNPs selection and genotyping

Two techniques for DNA collection were adopted. All participants were encouraged to provide a forearm venous blood sample, of whom 189 did so. If participants were unwilling to provide a blood sample or there was difficulty in obtaining a blood sample, a saliva sample was collected (116 participants). It was not possible to collect either sample from two participants. Genotyping was successful for 99.9% of the sample-SNPs combinations (7,313 out of 7,320); four different assays did not work for four samples, and three for one sample.

Blood (5 mL) was collected from a superficial forearm vein in 5-mL EDTA tubes (BD Vacutainer Systems, Plymouth, UK). Samples were stored at −20 °C until further processing. Superfluous saliva samples were collected using Oragene DNA OG-500 collection tubes (DNA Genotek Inc., Ontario, Canada) according to the manufacturer’s instructions. Saliva samples were stored at room temperature until DNA extraction. Genomic DNA was extracted from both blood and saliva samples using a QIAcube, QIAamp DNA Blood Mini kit and standard spin column protocol (Qiagen, Crawley, UK).

Extracted DNA samples were genotyped for 24 polymorphisms selected based on the literature on the previous associations of those SNPs with relevant phenotypes, previous or hypothesised associations with sarcopenia and understood functional relevance. An initially 36 candidate SNPs identified were then reduced to 24 considering the number of studies reporting each association, the presence of conflicting results and the known transcriptional difference for some of the SNPs. Associations of the SNPs with previously reported relevant phenotypes are presented in Supplementary Table S2.

Two techniques were adopted for genotyping. The Fluidigm EP1 system (Fluidigm, Cambridge, UK) was used initially, but where errors occurred (~1%), such as when duplicate samples were not in agreement, a second run was performed using StepOnePlus real-time PCR (Applied Biosystems^®^, Paisley, UK).In brief, four runs were performed, and genotype was determined using Fluidigm 192.24 Dynamic Array IFC (Integrated Fluidic Circuit, Fluidigm) in accordance with the manufacturer’s instructions. Each assay (4 µL) comprised 2.0 µL of assay loading reagent [2X] (Fluidigm), 1.0 µL SNP genotyping Assay Mix [40X] (Applied Biosystems^®^), 0.2 µL ROX [50X] (Invitrogen, Carlsbad, CA) and 0.8 µL DNA-free water (Qiagen). Each sample (4 µL) contained 1.6 µL genomic DNA, 2.0 µL GTXpress master mix [2X] (Applied Biosystems^®^, PN 4401892), 0.2 µL Fast GT Sample Loading Reagent [20X] (Fluidigm, PN 100–3065), and 0.2 µL DNA-free water. No-template controls (NTCs) were included in each run. Each of the assays (3.75 µL) and samples (4 µL) were pipetted into separate inlets of the chip according to manufacturer’s instructions. Assays and samples were loaded and mixed using the Integrated IFC Controller RX software. The chip was then loaded into a thermal cycler (FC1 Fluidigm, PN 100–1279 D1) and the GT 192.24 Fast v1.pcl protocol was run. The thermocycling protocol was: an initial 120 s at 95 °C followed by 45 cycles of denaturation for 2 s at 95 °C and then annealing and extension for 20 s at 60 °C. TaqMan assays included VIC^®^ and FAM^®^ dyes for all SNPs and genotypes were identified based on end-point fluorescence (https://www.thermofisher.com/np/en/home.html) (attached in Supplementary Table S3). When using the StepOnePlus, the reaction volume was 10 µL that contained 0.2 µL DNA, 5 µL GTXpress master mix, 4.3 µL nuclease-free H_2_O and 0.5 µL TaqMan SNP genotyping assay [20X]. An initial 20 s at 95 °C was followed by 50 cycles of denaturation for 3 s at 95 °C, then annealing and extension for 20 s at 60 °C. Genotypes were identified based on reporter dyes VIC^®^ and FAM^®^ intensity and visualized using cluster plots. Except for *PTK2* rs7460, for which nucleotides were reported on the reverse strand according to NCBI, others were reported on the forward strand (shown in Supplementary Table S3). All samples were analysed in duplicate and 100% agreement was required to minimise genotyping error^89^.

### Assessment of sarcopenia

Sarcopenia was assessed using three different definitions. The first definition, %SMM, was calculated as SMM/body mass*100. Participants were defined as sarcopenic if %SMM < 22.1%^27^. The second definition, SMI, previously used by Janssen *et al*.^28^, was calculated as SMM/height^2^. Participants were defined as sarcopenic if SMI ≤ 6.76 kg/m^2^ ^28^. Definition three used the measures of low SMI and low HGS as suggested by the EWGSOP; for which individuals with both SMI < 6.76 kg/m^2^ and a HGS < 20 kg were considered sarcopenic^1^.

### Statistical analysis

The Kolmogorov-Smirnov test was used to assess whether the data had a normal distribution and Levene’s test was used to assess the homogeneity of variance of HGS and SMI. The frequency distribution of each SNP was assessed for Hardy-Weinberg equilibrium (HWE) using chi-square tests. Binary logistic regression was performed to investigate the association of sarcopenia and the SNPs studied, with age and PASE score used as covariate. The analysis was performed separately for each individual SNP. In instances where the number of homozygous participants was low, the homozygous group was combined with the heterozygous group and a two-group analysis was performed. p < 0.05 was considered statistically significant. Where there was an association or tendency of an association (0.05 < p < 0.15)^90,91^, the homozygous groups were combined with the heterozygous group in a recessive and dominant model and then the analyses were re-run. Odds ratios (OR) for the risk genotype for sarcopenia were estimated for each SNP. Benjamini-Hochberg correction was performed to reduce the chance of type I error for multiple testing^92^ with 24 tests and False Discovery Rate of 0.25. All the tests were performed in SPSS Version 26.0.

### Ethics statement

Study protocols were in accordance with the guidelines of the Declaration of Helsinki (World Medical Association, 2013) and approved by the Ethics Committee of Manchester Metropolitan University. Informed written consent was obtained from all participants prior to involvement in the study.

## Supplementary information

Supplementary Information.

Supplementary dataset.

## Data Availability

The datasets analysed during the present study are available on reasonable request from corresponding author.

## References

[1] Cruz-Jentoft, A. J. *et al*. Sarcopenia: European consensus on definition and diagnosisReport of the European Working Group on Sarcopenia in Older People. *Age ageing* **39**, 412–423 (2010). 10.1093/ageing/afq034 PMC2886201 20392703

[2] McNamee P, Bond J, Buck D (2001). Resource Implications Study of the Medical Research Council Cognitive, F. & Ageing, S. Costs of dementia in England and Wales in the 21st century. Br. J. Psychiatry.

[3] Fahy N (2012). Who is shaping the future of European health systems?. BMJ.

[4] Cederholm T, Cruz-Jentoft AJ, Maggi S (2013). Sarcopenia and fragility fractures. Eur. J. Phys. Rehabil. Med..

[5] Dent, E. *et al*. International Clinical Practice Guidelines for Sarcopenia (ICFSR): Screening, Diagnosis and Management. *J. Nutr. Health Aging***22**, 1148–1161, 10.1007/s12603-018-1139-9 (2018).10.1007/s12603-018-1139-930498820

[6] Fielding RA (2011). Sarcopenia: an undiagnosed condition in older adults. Current consensus definition: prevalence, etiology, and consequences. International working group on sarcopenia. J. Am. Med. Dir. Assoc..

[7] Beaudart C (2015). Estimation of sarcopenia prevalence using various assessment tools. Exp. gerontology.

[8] Bijlsma AY (2013). Defining sarcopenia: the impact of different diagnostic criteria on the prevalence of sarcopenia in a large middle aged cohort. Age..

[9] Pagotto V, Silveira EA (2014). Methods, diagnostic criteria, cutoff points, and prevalence of sarcopenia among older people. Sci. World J..

[10] Isanejad M (2016). Dietary protein intake is associated with better physical function and muscle strength among elderly women. Br. J. Nutr..

[11] Steffl M (2017). Relationship between sarcopenia and physical activity in older people: a systematic review and meta-analysis. Clin. Interv. Aging.

[12] Carmelli D, Reed T (2000). Stability and change in genetic and environmental influences on hand-grip strength in older male twins. J. Appl. Physiol..

[13] de Camargo Smolarek A (2018). Strength Decline in Sedentary Males and Females of Different Ages. J. Exerc. Physiol. Online.

[14] Bruce S (2017). Healthy diet and better muscle function and quality in older women. Age Ageing.

[15] Gerdhem P, Ringsberg KAM, Obrant KJ, Akesson K (2005). Association between 25-hydroxy vitamin D levels, physical activity, muscle strength and fractures in the prospective population-based OPRA Study of Elderly Women. Osteoporos. Int..

[16] Charbonneau DE (2008). ACE genotype and the muscle hypertrophic and strength responses to strength training. Med. Sci. Sports Exerc..

[17] Garatachea N, Lucia A (2013). Genes, physical fitness and ageing. Ageing Res. Rev..

[18] Tan LJ, Liu SL, Lei SF, Papasian CJ, Deng HW (2012). Molecular genetic studies of gene identification for sarcopenia. Hum. Genet..

[19] Cho J, Lee I, Kang H (2017). ACTN3 Gene and Susceptibility to Sarcopenia and Osteoporotic Status in Older Korean Adults. Biomed. Res. Int..

[20] Roth SM, Zmuda JM, Cauley JA, Shea PR, Ferrell RE (2004). Vitamin D receptor genotype is associated with fat-free mass and sarcopenia in elderly men. J. Gerontology Ser. A: Biol. Sci. Med. Sci..

[21] Tasar PT (2018). Retrospective investigation of Interleukin IL-1 and IL-6 genes polymorphism among elderly patients with sarcopenia in the Turkish population. Genet. Mol. Res..

[22] Walsh S, Ludlow AT, Metter EJ, Ferrucci L, Roth SM (2016). Replication study of the vitamin D receptor (VDR) genotype association with skeletal muscle traits and sarcopenia. Aging Clin. Exp. Res..

[23] Tosun Tasar P (2015). Myostatin Gene Polymorphism in an Elderly Sarcopenic Turkish Population. Genet. Test. Mol. Biomarkers.

[24] Erskine, R. M., Jones, D. A., Maganaris, C. N. & Degens, H. *In vivo* specific tension of the human quadriceps femoris muscle. *Eur. J. Appl. Physiol.* **106**, 827, 10.1007/s00421-009-1085-7 (2009). 10.1007/s00421-009-1085-7 19468746

[25] Stebbings, G. K., Morse, C. I., Williams, A. G., Day, S. H. Variability and distribution of muscle strength and its determinants in humans. *Muscle Nerve* **49**, 879–886, 10.1002/mus.24075 (2014). 10.1002/mus.24075 24037782

[26] Chen, L., Nelson, D. R., Zhao, Y., Cui, Z. & Johnston, J. A. J. B. g. Relationship between muscle mass and muscle strength, and the impact of comorbidities: a population-based, cross-sectional study of older adults in the United States. BMC Geriatr **13**, 74, 10.1186/1471-2318-13-74 (2013). 10.1186/1471-2318-13-74 PMC3765109 23865675

[27] Janssen, I., Heymsfield, S. B. & Ross, R. Low relative skeletal muscle mass (sarcopenia) in older persons is associated with functional impairment and physical disability. *J. Am. Geriatrics Soc.* **50**, 889–896, 10.1046/j.1532-5415.2002.50216.x (2002). 10.1046/j.1532-5415.2002.50216.x 12028177

[28] Janssen I, Baumgartner RN, Ross R, Rosenberg IH, Roubenoff R (2004). Skeletal muscle cutpoints associated with elevated physical disability risk in older men and women. Am. J. Epidemiol..

[29] Tichet J (2008). Prevalence of sarcopenia in the French senior population. J. Nutr. Health Aging.

[30] Chien MY, Huang TY, Wu YT (2008). Prevalence of sarcopenia estimated using a bioelectrical impedance analysis prediction equation in community‐dwelling elderly people in Taiwan. J. Am. Geriatrics Soc..

[31] Janssen I (2006). Influence of sarcopenia on the development of physical disability: the Cardiovascular Health Study. J. Am. Geriatr. Soc..

[32] Wu CH (2014). Prevalence and associated factors of sarcopenia and severe sarcopenia in older T aiwanese living in rural community: The T ianliao O ld P eople study 04. Geriatrics gerontology Int..

[33] Kemmler W (2015). Prevalence of sarcopenia in Germany and the corresponding effect of osteoarthritis in females 70 years and older living in the community: results of the FORMoSA study. Clin. Interv. Aging.

[34] Yoshida D (2014). Using two different algorithms to determine the prevalence of sarcopenia. Geriatrics gerontology Int..

[35] Volpato S (2014). Prevalence and clinical correlates of sarcopenia in community-dwelling older people: application of the EWGSOP definition and diagnostic algorithm. J. Gerontol. A Biol. Sci. Med. Sci.

[36] Yamada M (2013). Prevalence of sarcopenia in community-dwelling Japanese older adults. J. Am. Med. Dir. Assoc..

[37] Arango-Lopera VE, Arroyo P, Gutiérrez-Robledo LM, Pérez-Zepeda MU (2012). Prevalence of sarcopenia in Mexico City. Eur. Geriatric Med..

[38] ter Borg S (2016). Differences in nutrient intake and biochemical nutrient status between sarcopenic and nonsarcopenic older adults—results from the Maastricht Sarcopenia Study. J. Am. Med. Dir. Assoc..

[39] Velázquez Alva MDC, Irigoyen Camacho ME, Delgadillo Velázquez J, Lazarevich I (2013). The relationship between sarcopenia, undernutrition, physical mobility and basic activities of daily living in a group of elderly women of Mexico City. Nutricion hospitalaria.

[40] Kim KM, Jang HC, Lim SJTKjoim (2016). Differences among skeletal muscle mass indices derived from height-, weight-, and body mass index-adjusted models in assessing sarcopenia. Korean J. Intern. Med..

[41] Sonestedt E (2011). Association between fat intake, physical activity and mortality depending on genetic variation in FTO. Int. J. Obes..

[42] Livshits G, Malkin I, Moayyeri A, Spector TD, Hammond CJ (2012). Association of FTO gene variants with body composition in UK twins. Ann. Hum. Genet..

[43] Frayling TM (2007). A common variant in the FTO gene is associated with body mass index and predisposes to childhood and adult obesity. Sci..

[44] Sabarneh A (2018). Common FTO rs9939609 variant and risk of type 2 diabetes in Palestine. BMC Med. Genet..

[45] Feng Y (2014). Obesity-associated gene FTO rs9939609 polymorphism in relation to the risk of tuberculosis. BMC Infect. Dis..

[46] Wang X (2017). FTO is required for myogenesis by positively regulating mTOR-PGC-1α pathway-mediated mitochondria biogenesis. Cell death Dis..

[47] Jess T (2008). Impact on weight dynamics and general growth of the common FTO rs9939609: a longitudinal Danish cohort study. Int. J. Obes..

[48] Guilherme JPL (2019). The A-allele of the FTO Gene rs9939609 Polymorphism Is Associated With Decreased Proportion of Slow Oxidative Muscle Fibers and Over-represented in Heavier Athletes. J. Strength. Conditioning Res..

[49] Heffernan SM (2017). Fat mass and obesity associated (FTO) gene influences skeletal muscle phenotypes in non-resistance trained males and elite rugby playing position. BMC Genet..

[50] Eider J (2014). Endothelial nitric oxide synthase g894t (rs1799983) gene polymorphism in polish athletes. Open. Life Sci..

[51] Zmijewski P, Cieszczyk P, Ahmetov II (2018). The NOS3 G894T (rs1799983) and-786T/C (rs2070744) polymorphisms are associated with elite swimmer status. Biol. Sport..

[52] Guidry, M. A. *et al*. Endothelial Nitric Oxide Synthase (NOS3). *ISRN Vascular Medicine* **2012** (2012).

[53] Bray MS (2009). The human gene map for performance and health-related fitness phenotypes: the 2006-2007 update. Med. Sci. Sports Exerc..

[54] Brown GC (2007). Mechanisms of inflammatory neurodegeneration: iNOS and NADPH oxidase. Biochem. Soc. Trans..

[55] Smith LW, Smith JD, Criswell DS (2002). Involvement of nitric oxide synthase in skeletal muscle adaptation to chronic overload. J. Appl. Physiol..

[56] Persu A (2002). Modifier effect of ENOS in autosomal dominant polycystic kidney disease. Hum. Mol. Genet..

[57] Lemoine S (2003). Estrogen receptor alpha mRNA in human skeletal muscles. Med. Sci. Sports Exerc..

[58] Luo L (2014). Association of ESR1 and C6orf97 gene polymorphism with osteoporosis in postmenopausal women. Mol. Biol. Rep..

[59] Martinaityte I (2017). Bone mineral density is associated with vitamin D related rs6013897 and estrogen receptor polymorphism rs4870044: The Tromsø study. PLoS one.

[60] Hidalgo-Bravo A (2019). Association of RMND1/CCDC170-ESR1 single nucleotide polymorphisms with hip fracture and osteoporosis in postmenopausal women. Climacteric.

[61] Daly R, Saxon L, Turner C, Robling A, Bass SJB (2004). The relationship between muscle size and bone geometry during growth and in response to exercise. Bone.

[62] Klein CS, Allman BL, Marsh GD, Rice CL (2002). Muscle size, strength, and bone geometry in the upper limbs of young and old men. J. Gerontol. A Biol. Sci. Med. Sci.

[63] Karasik D (2009). Bivariate genome-wide linkage analysis of femoral bone traits and leg lean mass: Framingham study. J. Bone Min. Res..

[64] Ronkainen PH (2008). Catechol-o-methyltransferase gene polymorphism is associated with skeletal muscle properties in older women alone and together with physical activity. PLoS One.

[65] Salmen T (2002). Relation of estrogen receptor-alpha gene polymorphism and hormone replacement therapy to fall risk and muscle strength in early postmenopausal women. Ann. Med..

[66] Vandevyver C (1999). Lack of association between estrogen receptor genotypes and bone mineral density, fracture history, or muscle strength in elderly women. J. Bone Min. Res..

[67] Bjornstrom L, Sjoberg M (2005). Mechanisms of estrogen receptor signaling: convergence of genomic and nongenomic actions on target genes. Mol. Endocrinol..

[68] Yasar P, Ayaz G, User SD, Gupur G, Muyan M (2017). Molecular mechanism of estrogen-estrogen receptor signaling. Reprod. Med. Biol..

[69] Cauley JA (2015). Estrogen and bone health in men and women. Steroids.

[70] Gennari L (2005). Estrogen receptor gene polymorphisms and the genetics of osteoporosis: a HuGE review. Am. J. Epidemiol..

[71] Rolland YM, Perry HM, Patrick P, Banks WA, Morley JE (2007). Loss of appendicular muscle mass and loss of muscle strength in young postmenopausal women. J. Gerontol. A Biol. Sci. Med. Sci.

[72] Phillips SK, Sanderson AG, Birch K, Bruce SA, Woledge RC (1996). Changes in maximal voluntary force of human adductor pollicis muscle during the menstrual cycle. J. Physiol..

[73] Liu X-G (2009). Genome-wide association and replication studies identified TRHR as an important gene for lean body mass. Am. J. Hum. Genet..

[74] Miyamoto-Mikami E (2017). Lack of association between genotype score and sprint/power performance in the Japanese population. J. Sci. Med. sport..

[75] Fuku N (2015). Exceptional longevity and muscle and fitness related genotypes: a functional *in vitro* analysis and case-control association replication study with SNPs THRH rs7832552, IL6 rs1800795, and ACSL1 rs6552828. Front. Aging Neurosci..

[76] Salvatore D, Simonides WS, Dentice M, Zavacki AM, Larsen PR (2014). Thyroid hormones and skeletal muscle—new insights and potential implications. Nat. Rev. Endocrinol..

[77] Janssen I, Heymsfield SB, Baumgartner RN, Ross R (2000). Estimation of skeletal muscle mass by bioelectrical impedance analysis. J. Appl. Physiol..

[78] Faria SL, Faria OP, Cardeal MD, Ito MKJOs (2014). Validation study of multi-frequency bioelectrical impedance with dual-energy X-ray absorptiometry among obese patients. Obes. Surg..

[79] Okasora K (1999). Comparison of bioelectrical impedance analysis and dual energy X-ray absorptiometry for assessment of body composition in children. Pediatr. Int..

[80] Lee S (2018). Comparison between dual-energy X-ray absorptiometry and bioelectrical impedance analyses for accuracy in measuring whole body muscle mass and appendicular skeletal muscle mass. Nutrients.

[81] Wingo BC, Barry VG, Ellis AC, Gower BA (2018). Comparison of segmental body composition estimated by bioelectrical impedance analysis and dual-energy X-ray absorptiometry. Clin. Nutr. ESPEN.

[82] Barrea, L. *et al*. Association between Mediterranean diet and hand grip strength in older adult women. **38**, 721–729 (2019). 10.1016/j.clnu.2018.03.012 29643004

[83] Hughes DC, Day SH, Ahmetov II, Williams AG (2011). Genetics of muscle strength and power: polygenic profile similarity limits skeletal muscle performance. J. Sports Sci..

[84] Lee W-J (2013). Comparisons of sarcopenia defined by IWGS and EWGSOP criteria among older people: results from the I-Lan longitudinal aging study. J. Am. Med. Dir. Assoc..

[85] Wang H (2016). Estimation of prevalence of sarcopenia by using a new bioelectrical impedance analysis in Chinese community-dwelling elderly people. BMC geriatrics.

[86] Roberts HC (2011). A review of the measurement of grip strength in clinical and epidemiological studies: towards a standardised approach. Age Ageing.

[87] Villafane JH (2016). Reliability of the Handgrip Strength Test in Elderly Subjects With Parkinson Disease. Hand.

[88] Washburn, R., Ficker, J. J. J. O. S. M. & Fitness, P. Physical Activity Scale for the Elderly (PASE): the relationship with activity measured by a portable accelerometer. **39**, 336 (1999). 10726435

[89] Tintle N, Gordon D, Van Bruggen D, Finch S (2009). The cost effectiveness of duplicate genotyping for testing genetic association. Ann. Hum. Genet..

[90] Danilovic DL (2007). Height and bone mineral density in androgen insensitivity syndrome with mutations in the androgen receptor gene. Osteoporos. Int..

[91] Fischer CP (2004). Endurance training reduces the contraction-induced interleukin-6 mRNA expression in human skeletal muscle. Am. J. Physiol. Endocrinol. Metab..

[92] Benjamini, Y. & Hochberg, Y. Controlling the false discovery rate: a practical and powerful approach to multiple testing. *Journal of the royal statistical society. Series B (Methodological)*, 289–300 (1995).

