# Supplementary material for: Prevalence and association of single nucleotide polymorphisms with sarcopenia in older women depends on definition
Source: Sci Rep. 2020 Feb 19;10:2913. doi: 10.1038/s41598-020-59722-9 (PMC7031370; doi:10.1038/s41598-020-59722-9)
Supplement: Supplementary file 2 — Supplementary dataset. [file 41598_2020_59722_MOESM2_ESM.docx]

Title: Prevalence and association of single nucleotide polymorphisms with sarcopenia in older women depends on definition

Praval Khanal, Lingxiao He, Georgina Stebbings, Gladys L Onambele-Pearson, Hans Degens, Alun Williams, Martine Thomis, Christopher I Morse

Table S1. Genotype frequency and Hardy Weinberg Equilibrium

| SNPs | Frequency | χ² p-value | |
| --- | --- | --- | --- |
| *IL6* rs1800795  CC (n=61)  CG (n=145)  GG (n=99) | 20.0%  47.5%  32.5% | 0.356 | 0.837 |
| *FTO* rs9939609  AA (n=48)  AT (n=151)  TT (n=106) | 15.7%  49.5%  34.8% | 0.228 | 0.892 |
| *MTHFR* rs17421511  AA (n=7)  AG (n=78)  GG (n=220) | 2.3%  25.6%  72.1% | 0.001 | 0.99 |
| *ACVR1B* rs10783485  GG (n=128)  GT (n=147)  TT (n=29) | 42.1%  48.4%  9.5% | 2.036 | 0.361 |
| *NOS3* rs1799983  GG (n=118)  GT (n=144)  TT (n=43) | 38.7%  47.2%  14.1% | 0.008 | 0.996 |
| *ACVR1B* rs2854464  AA (n=153)  AG (n=128)  GG (n=24) | 50.2%  42.0%  7.8% | 0.150 | 0.928 |
| *PTK2* rs7460  TT (n=72)  AT (n=153)  AA (n=80) | 23.6%  50.2%  26.2% | 0.005 | 0.998 |
| *ESR1* rs1999805  AA (n=100)  AG (n=148)  GG (n=57) | 32.8%  48.5%  18.7% | 0.029 | 0.985 |
| *PTK2* rs7843014  AA ((n=105)  AC (n=142)  CC (n=57) | 34.5%  46.7%  18.8% | 0.534 | 0.766 |
| *VDR* rs2228570  AA (n=103)  AG (n= 154)  GG (n=48) | 33.8%  50.5%  15.7% | 0.585 | 0.747 |
| *ID3* rs11574  CC (n=178)  CT (n=111)  TT (n=16) | 58.4%  36.4%  5.2% | 0.059 | 0.971 |
| *CNTF* rs1800169  AA (n=3)  AG (n=77)  GG (n=225) | 1.0%  25.2%  73.8% | 1.662 | 0.436 |
| *ACE* rs4341  CC (n=63)  CG (n=142)  GG (n=100) | 20.7%  46.5%  32.8% | 0.921 | 0.631 |
| *CNTFR* rs2070802  AA (n=216)  AT (n=79)  TT (n=10) | 70.8%  25.9%  3.3% | 0.686 | 0.710 |
| *MTHFR* rs1801131  GG (n=27)  GT (n=133)  TT (n=144) | 8.9%  43.8%  47.3% | 0.224 | 0.894 |
| *ESR1* rs4870044  CC (n=158)  CT (n=122)  TT (n=24) | 52.0%  40.1%  7.9% | 0.004 | 0.998 |
| *COL1A1* rs1800012  AA (n=10)  AC (n=89)  CC (n=205) | 3.3%  29.3%  67.4% | 0.008 | 0.996 |
| *ACTN3* rs1815739  CC (n=103)  CT (n=133)  TT (n=68) | 33.9%  43.8%  22.4% | 3.899 | 0.142 |
| *HIF1A* rs11549465  CC (n=241)  CT (n=62)  TT (n=2) | 79.0%  20.3%  0.7% | 0.869 | 0.648 |
| *MSTN* rs1805086  CC (n=0)  CT (n=10)  TT (n=295) | 0.0%  3.3%  96.7% | 0.085 | 0.959 |
| *MTHFR* rs1537516  AA (n=4)  AG (n=56)  GG (n=245) | 1.3%  18.4%  80.3% | 0.154 | 0.926 |
| *TTN* rs10497520  CC (n=235)  CT (n=66)  TT (n=4) | 77.1%  21.6%  1.3% | 0.069 | 0.966 |
| *TRHR* rs7832552  CC (n=137)  CT (n=130)  TT (n=37) | 45.0%  42.8%  12.2% | 0.510 | 0.775 |
| *IGF1* rs35767  AA (n=4)  AG (n=76)  GG (n=225) | 1.3%  24.9%  73.8% | 0.740 | 0.691 |

Table S2. Previous associations of single nucleotide polymorphisms/gene product and muscle related phenotypes/performance

| Single nucleotide polymorphisms | Muscle phenotypes studied/physical performance | Main results | References |
| --- | --- | --- | --- |
| *ACTN3* rs1815739 | Lower mid-thigh cross sectional area  KE shortening and lengthening peak torque  Sarcopenia | XX demonstrated significantly lower than RX/RR  XX group had significantly reduced than RR/RX groups  XX as risk group for sarcopenia | (Zempo et al., 2010)  (Walsh et al., 2008)  (Cho et al., 2017) |
| *ACE* rs4341  (I/D) | Lean mass, and body weight; appendicular fat free mass in older women  Handgrip strength and vertical jump ability in adolescents | Higher body mass measures were associated with D allele  II genotype is associated with higher strength and jump performance | (Charbonneau et al., 2008)    (Moran et al., 2006) |
| *CNTF* rs1800169 | Concentric peak torque knee extensors and elbow flexors  Concentric knee flexors strength in middle aged women  Handgrip strength | Heterozygotes GA were stronger than GG individuals  A-allele carriers were weaker than GG  AA homozygotes had 3.8 kg weaker handgrip strength than G-allele carriers. | (Roth et al., 2001)  (De Mars et al., 2007)  (Arking et al., 2006) |
| *CNTFR* rs2070802 | 60-78 years male | T-allele carriers possessed higher Knee extension and knee flexion in male | (De Mars et al., 2007) |
| *ESR1*  rs1999805  rs4870044 | Lean mass  Bone Mineral Density (BMD) | Loss of oestrogen after menopause was associated with low lean mass  ESR1 rs4870044 TT genotype was associated with low BMD | (Poehlman et al., 1995)  (Luo et al., 2014) |
| *FTO* rs9939609 | BMI  Calf circumference | AA was associated with higher BMI, muscle mass, and obesity related phenotypes | (Jacobsson et al., 2012, Al-Serri et al., 2018) |
| *HIF1A* rs11549465 | Maximal oxygen consumption with exercise training in elderly  Frequency distribution of genotypes | TT was associated with higher V02 max  TT genotype was over represented in weightlifters and wrestlers compared to controls | (Prior et al., 2003)  (Ahmetov et al., 2008) |
| *ID3* rs11574 | Fat mass, BMI, waist circumference (WC), and waist‐hip ratio (WHR) in humans | A-allele was associated with changes in cross sectional BMI and fat mass | (Svendstrup et al., 2018) |
| *IGF1* rs35767 | Body composition | CC genotype was associated with higher trunk and total fat and lower lean and muscle mass | (Kostek et al., 2010) |
| *IL6* rs1800795 | Weightlifters and jumpers  Knee muscle strength and frailty  Exceptional longevity | G allele was overrepresented  No association  No association | (Ruiz et al., 2010)  (Walston et al., 2005, Pereira et al., 2011)  (Fuku et al., 2015) |
| *MTHFR*  rs1801131  rs1537516  17421511 | Sprint and strength athletes  VO_2_max post training | *MTHFR* rs1801131 C-allele overrepresentation  C-allele carriers had significantly improvement in VO_2_ Max | (Zarebska et al., 2014)  (Cięszczyk et al., 2016) |
| *PTK2*  rs7843014,  rs7460 | Exceptional longevity  Specific force in healthy men | rs7843014 CC and rs7460 TT association with longevity  AA homozygotes had higher VL specific force | (Garatachea et al., 2014)  (Erskine et al., 2012, Stebbings et al., 2017) |
| *TRHR* rs7832552 | Lean body mass  Exceptional longevity | T allele was associated with higher lean body mass  No association | (Liu et al., 2009, Lunardi et al., 2013)  (Fuku et al., 2015) |
| *TTN* rs10497520 | Marathon running performance and muscle fascicle length  Isometric knee strength in CAD patients  Isometric knee extension | T-allele carriers had better marathon personal best times.  No association with strength  C-allele is predisposing allele for knee strength in elderly population | (Stebbings et al., 2018)  (Thomaes et al., 2013)  (He et al., 2018) |
| *VDR* rs2228570 | Quadriceps strength, hamstring strength, peak torque, FFM  Knee strength  FFM/Sarcopenia | F (C) allele has been linked to reduced fat-free-mass and men  F allele with reduced concentric and isometric knee strength than f-allele carriers  FF homozygotes in higher risk of sarcopenia than f-allele carriers and f allele associated with higher FFM | (Roth et al., 2004)  (Windelinckx et al., 2007)  (Roth et al., 2004, Walsh et al., 2016) |
| *MSTN* rs1805086 | Muscle strength/muscle size | R153 allele of MSTN with decreased strength  A negative influence of 1RM leg press and muscle mass of women at old age  R allele linked to maximal isometric contraction of elbow muscle flexors and ability to produce peak power during muscle contractions  No association  KR has significantly higher increment in bicep and quadriceps thickness than KK genotypes | (Seibert et al., 2001, Corsi et al., 2002)  (González-Freire et al., 2010)  (Kostek et al., 2009, Santiago et al., 2011)  (Fuku et al., 2015)  (Li et al., 2014, Santiago et al., 2011) |
| *COL1A1* rs1800012 | Hand grip strength and biceps strength in elderly 70+ population | s allele is associated with the lower strength | (Van Pottelbergh et al., 2001) |
| *ACVR1B*  rs2854464  *ACVR1B*  rs10783485 |  | rs2854464 A allele associated with higher knee strength  over representation of A allele in sprint and power Caucasians athletes  A-allele with SMM  C-allele as strength increasing allele for isometric knee flexion | (Windelinckx et al., 2011)  (Voisin et al., 2016)  (He et al., 2018)  (Windelinckx et al., 2011) |
| *NOS3* rs1799983 | Distribution in athletic population and long distance swimmers  Stroke volume | T allele was overrepresented in the power athletes  T-allele was associated with higher stroke volume and lower HR during submaximal dynamic exercise in postmenopausal women | (Gómez-Gallego et al., 2009, Sessa et al., 2011, Zmijewski et al., 2018, Eider et al., 2014)  (Hand et al., 2006) |

AHMETOV, II, HAKIMULLINA, A. M., LYUBAEVA, E. V., VINOGRADOVA, O. L. & ROGOZKIN, V. A. 2008. Effect of HIF1A gene polymorphism on human muscle performance. *Bulletin of experimental biology and medicine,* 146**,** 351-353.

AL-SERRI, A., AL-BUSTAN, S. A., KAMKAR, M., THOMAS, D., ALSMADI, O., AL-TEMAIMI, R., MOJIMINIYI, O. A. & ABDELLA, N. A. 2018. Association of FTO rs9939609 with Obesity in the Kuwaiti Population: A Public Health Concern? *Medical Principles and Practice,* 27**,** 145-151.

ARKING, D. E., FALLIN, D. M., FRIED, L. P., LI, T., BEAMER, B. A., XUE, Q. L., CHAKRAVARTI, A. & WALSTON, J. 2006. Variation in the ciliary neurotrophic factor gene and muscle strength in older Caucasian women. *Journal of the American Geriatrics Society,* 54**,** 823-826.

CHARBONNEAU, D. E., HANSON, E. D., LUDLOW, A. T., DELMONICO, M. J., HURLEY, B. F. & ROTH, S. M. 2008. ACE genotype and the muscle hypertrophic and strength responses to strength training. *Medicine and science in sports and exercise,* 40**,** 677.

CHO, J., LEE, I. & KANG, H. 2017. ACTN3 gene and susceptibility to sarcopenia and osteoporotic status in older Korean adults. *BioMed research international,* 2017.

CIĘSZCZYK, P., ZARĘBSKA, A., JASTRZĘBSKI, Z., SAWCZYN, M., KOZAKIEWICZ-DROBNIK, I., LEOŃSKA-DUNIEC, A., KACZMARCZYK, M., MACIEJEWSKA-SKRENDO, A., ŻMIJEWSKI, P. & TRYBEK, G. 2016. Does the MTHFR A1298C Polymorphism Modulate the Cardiorespiratory Response to Training? *Journal of human kinetics,* 54**,** 43-53.

CORSI, A. M., FERRUCCI, L., GOZZINI, A., TANINI, A. & BRANDI, M. L. 2002. Myostatin polymorphisms and age‐related sarcopenia in the Italian population. *Journal of the American Geriatrics Society,* 50**,** 1463-1463.

DE MARS, G., WINDELINCKX, A., BEUNEN, G., DELECLUSE, C., LEFEVRE, J. & THOMIS, M. A. I. 2007. Polymorphisms in the CNTF and CNTF receptor genes are associated with muscle strength in men and women. *Journal of Applied Physiology,* 102**,** 1824-1831.

EIDER, J., FICEK, K., KACZMARCZYK, M., MACIEJEWSKA-KARŁOWSKA, A., SAWCZUK, M. & CIĘSZCZYK, P. 2014. Endothelial nitric oxide synthase g894t (rs1799983) gene polymorphism in polish athletes. *Open Life Sciences,* 9**,** 260-267.

ERSKINE, R. M., WILLIAMS, A. G., JONES, D. A., STEWART, C. E. & DEGENS, H. 2012. Do PTK2 gene polymorphisms contribute to the interindividual variability in muscle strength and the response to resistance training? A preliminary report. *Journal of applied physiology,* 112**,** 1329-1334.

FUKU, N., HE, Z.-H., SANCHIS-GOMAR, F., PAREJA-GALEANO, H., TIAN, Y., ARAI, Y., ABE, Y., MURAKAMI, H., MIYACHI, M. & ZEMPO, H. 2015. Exceptional longevity and muscle and fitness related genotypes: a functional in vitro analysis and case-control association replication study with SNPs THRH rs7832552, IL6 rs1800795, and ACSL1 rs6552828. *Frontiers in aging neuroscience,* 7**,** 59.

GARATACHEA, N., FUKU, N., HE, Z.-H., TIAN, Y., ARAI, Y., ABE, Y., MURAKAMI, H., MIYACHI, M., YVERT, T. & VENTURINI, L. 2014. PTK2 rs7460 and rs7843014 polymorphisms and exceptional longevity: a functional replication study. *Rejuvenation research,* 17**,** 430-438.

GÓMEZ-GALLEGO, F., RUIZ, J. R., BUXENS, A., ARTIEDA, M., ARTETA, D., SANTIAGO, C., RODRÍGUEZ-ROMO, G., LAO, J. I. & LUCIA, A. 2009. The− 786 T/C polymorphism of the NOS3 gene is associated with elite performance in power sports. *European journal of applied physiology,* 107**,** 565-569.

GONZÁLEZ-FREIRE, M., RODRÍGUEZ-ROMO, G., SANTIAGO, C., BUSTAMANTE-ARA, N., YVERT, T., GÓMEZ-GALLEGO, F., REXACH, J. A. S., RUIZ, J. R. & LUCIA, A. 2010. The K153R variant in the myostatin gene and sarcopenia at the end of the human lifespan. *Age,* 32**,** 405-409.

HAND, B. D., MCCOLE, S. D., BROWN, M. D., PARK, J. J., FERRELL, R. E., HUBERTY, A., DOUGLASS, L. W. & HAGBERG, J. M. 2006. NOS3 gene polymorphisms and exercise hemodynamics in postmenopausal women. *International journal of sports medicine,* 27**,** 951-958.

HE, L., VAN ROIE, E., BOGAERTS, A., MORSE, C. I., DELECLUSE, C., VERSCHUEREN, S. & THOMIS, M. 2018. Genetic predisposition score predicts the increases of knee strength and muscle mass after one-year exercise in healthy elderly. *Experimental gerontology,* 111**,** 17-26.

JACOBSSON, J. A., SCHIÖTH, H. B. & FREDRIKSSON, R. 2012. The impact of intronic single nucleotide polymorphisms and ethnic diversity for studies on the obesity gene FTO. *Obesity Reviews,* 13**,** 1096-1109.

KOSTEK, M. A., ANGELOPOULOS, T. J., CLARKSON, P. M., GORDON, P. M., MOYNA, N. M., VISICH, P. S., ZOELLER, R. F., PRICE, T. B., SEIP, R. L. & THOMPSON, P. D. 2009. Myostatin and follistatin polymorphisms interact with muscle phenotypes and ethnicity. *Medicine and science in sports and exercise,* 41**,** 1063.

KOSTEK, M. C., DEVANEY, J. M., GORDISH-DRESSMAN, H., HARRIS, T. B., THOMPSON, P. D., CLARKSON, P. M., ANGELOPOULOS, T. J., GORDON, P. M., MOYNA, N. M. & PESCATELLO, L. S. 2010. A polymorphism near IGF1 is associated with body composition and muscle function in women from the Health, Aging, and Body Composition Study. *European journal of applied physiology,* 110**,** 315-324.

LI, X., WANG, S.-J., TAN, S. C., CHEW, P. L., LIU, L., WANG, L., WEN, L. & MA, L. 2014. The A55T and K153R polymorphisms of MSTN gene are associated with the strength training-induced muscle hypertrophy among Han Chinese men. *Journal of sports sciences,* 32**,** 883-891.

LIU, X.-G., TAN, L.-J., LEI, S.-F., LIU, Y.-J., SHEN, H., WANG, L., YAN, H., GUO, Y.-F., XIONG, D.-H. & CHEN, X.-D. 2009. Genome-wide association and replication studies identified TRHR as an important gene for lean body mass. *The American Journal of Human Genetics,* 84**,** 418-423.

LUNARDI, C. C., LIMA, R. M., PEREIRA, R. W., LEITE, T. K. M., SIQUEIRA, A. B. M. & OLIVEIRA, R. J. 2013. Association between polymorphisms in the TRHR gene, fat-free mass, and muscle strength in older women. *Age,* 35**,** 2477-2483.

LUO, L., XIA, W., NIE, M., SUN, Y., JIANG, Y., ZHAO, J., HE, S. & XU, L. J. M. B. R. 2014. Association of ESR1 and C6orf97 gene polymorphism with osteoporosis in postmenopausal women. 41**,** 3235-3243.

MORAN, C. N., VASSILOPOULOS, C., TSIOKANOS, A., JAMURTAS, A. Z., BAILEY, M. E. S., MONTGOMERY, H. E., WILSON, R. H. & PITSILADIS, Y. P. 2006. The associations of ACE polymorphisms with physical, physiological and skill parameters in adolescents. *European journal of human genetics,* 14**,** 332.

PEREIRA, D. S., GARCIA, D. M., NARCISO, F. M. S., SANTOS, M., DIAS, J. M. D., QUEIROZ, B. Z., SOUZA, E. R., NOBREGA, O. T. & PEREIRA, L. S. M. 2011. Effects of 174 G/C polymorphism in the promoter region of the interleukin-6 gene on plasma IL-6 levels and muscle strength in elderly women. *Brazilian journal of medical and biological research,* 44**,** 123-129.

POEHLMAN, E. T., TOTH, M. J. & GARDNER, A. W. 1995. Article RETRACTED: Changes in energy balance and body composition at menopause: A controlled longitudinal study. *Annals of internal medicine,* 123**,** 673-675.

PRIOR, S. J., HAGBERG, J. M., PHARES, D. A., BROWN, M. D., FAIRFULL, L., FERRELL, R. E. & ROTH, S. M. 2003. Sequence variation in hypoxia-inducible factor 1α (HIF1A): association with maximal oxygen consumption. *Physiological genomics,* 15**,** 20-26.

ROTH, S. M., SCHRAGER, M. A., FERRELL, R. E., RIECHMAN, S. E., METTER, E. J., LYNCH, N. A., LINDLE, R. S. & HURLEY, B. F. 2001. CNTF genotype is associated with muscular strength and quality in humans across the adult age span. *Journal of applied physiology,* 90**,** 1205-1210.

ROTH, S. M., ZMUDA, J. M., CAULEY, J. A., SHEA, P. R. & FERRELL, R. E. 2004. Vitamin D receptor genotype is associated with fat-free mass and sarcopenia in elderly men. *The Journals of Gerontology Series A: Biological Sciences and Medical Sciences,* 59**,** B10-B15.

RUIZ, J. R., BUXENS, A., ARTIEDA, M., ARTETA, D., SANTIAGO, C., RODRÍGUEZ-ROMO, G., LAO, J. I., GÓMEZ-GALLEGO, F. & LUCIA, A. 2010. The− 174 G/C polymorphism of the IL6 gene is associated with elite power performance. *Journal of science and medicine in sport,* 13**,** 549-553.

SANTIAGO, C., RUIZ, J. R., RODRÍGUEZ-ROMO, G., FIUZA-LUCES, C., YVERT, T., GONZALEZ-FREIRE, M., GÓMEZ-GALLEGO, F., MORÁN, M. & LUCIA, A. 2011. The K153R polymorphism in the myostatin gene and muscle power phenotypes in young, non-athletic men. *PloS one,* 6**,** e16323.

SEIBERT, M. J., XUE, Q. L., FRIED, L. P. & WALSTON, J. D. 2001. Polymorphic variation in the human myostatin (GDF‐8) gene and association with strength measures in the women's health and aging study II cohort. *Journal of the American Geriatrics Society,* 49**,** 1093-1096.

SESSA, F., CHETTA, M., PETITO, A., FRANZETTI, M., BAFUNNO, V., PISANELLI, D., SARNO, M., IUSO, S. & MARGAGLIONE, M. 2011. Gene polymorphisms and sport attitude in Italian athletes. *Genetic testing and molecular biomarkers,* 15**,** 285-290.

STEBBINGS, G. K., WILLIAMS, A. G., HERBERT, A. J., LOCKEY, S. J., HEFFERNAN, S. M., ERSKINE, R. M., MORSE, C. I. & DAY, S. H. 2018. TTN genotype is associated with fascicle length and marathon running performance. *Scandinavian journal of medicine & science in sports,* 28**,** 400-406.

STEBBINGS, G. K., WILLIAMS, A. G., MORSE, C. I. & DAY, S. H. 2017. Polymorphisms in PTK2 are associated with skeletal muscle specific force: an independent replication study. *European journal of applied physiology,* 117**,** 713-720.

SVENDSTRUP, M., APPEL, E. V. R., SANDHOLT, C. H., AHLUWALIA, T. S., ÄNGQUIST, L. H., THUESEN, B. H., JØRGENSEN, M. E., PEDERSEN, O., GRARUP, N. & HANSEN, T. 2018. Prospective studies exploring the possible impact of an ID3 polymorphism on changes in obesity measures. *Obesity,* 26**,** 747-754.

THOMAES, T., THOMIS, M., ONKELINX, S., GOETSCHALCKX, K., FAGARD, R., LAMBRECHTS, D. & VANHEES, L. 2013. Genetic predisposition scores associate with muscular strength, size and trainability. *Medicine and science in sports and exercise,* 45**,** 1451-1459.

VAN POTTELBERGH, I., GOEMAERE, S., NUYTINCK, L., DE PAEPE, A. & KAUFMAN, J. M. 2001. Association of the type I collagen alpha1 Sp1 polymorphism, bone density and upper limb muscle strength in community-dwelling elderly men. *Osteoporosis International,* 12**,** 895-901.

VOISIN, S., GUILHERME, J. P. F. L., YAN, X., PUSHKAREV, V. P., CIESZCZYK, P., MASSIDDA, M., CALÒ, C. M., DYATLOV, D. A., KOLUPAEV, V. A. & PUSHKAREVA, Y. E. 2016. ACVR1B rs2854464 is associated with sprint/power athletic status in a large cohort of Europeans but not Brazilians. *PloS one,* 11**,** e0156316.

WALSH, S., LIU, D., METTER, E. J., FERRUCCI, L. & ROTH, S. M. 2008. ACTN3 genotype is associated with muscle phenotypes in women across the adult age span. *Journal of Applied Physiology,* 105**,** 1486-1491.

WALSH, S., LUDLOW, A. T., METTER, E. J., FERRUCCI, L. & ROTH, S. M. 2016. Replication study of the vitamin D receptor (VDR) genotype association with skeletal muscle traits and sarcopenia. *Aging clinical and experimental research,* 28**,** 435-442.

WALSTON, J., ARKING, D. E., FALLIN, D., LI, T., BEAMER, B., XUE, Q., FERRUCCI, L., FRIED, L. P. & CHAKRAVARTI, A. 2005. IL-6 gene variation is not associated with increased serum levels of IL-6, muscle, weakness, or frailty in older women. *Experimental gerontology,* 40**,** 344-352.

WINDELINCKX, A., DE MARS, G., BEUNEN, G., AERSSENS, J., DELECLUSE, C., LEFEVRE, J. & THOMIS, M. A. I. 2007. Polymorphisms in the vitamin D receptor gene are associated with muscle strength in men and women. *Osteoporosis International,* 18**,** 1235-1242.

WINDELINCKX, A., DE MARS, G., HUYGENS, W., PEETERS, M. W., VINCENT, B., WIJMENGA, C., LAMBRECHTS, D., DELECLUSE, C., ROTH, S. M. & METTER, E. J. 2011. Comprehensive fine mapping of chr12q12-14 and follow-up replication identify activin receptor 1B (ACVR1B) as a muscle strength gene. *European Journal of Human Genetics,* 19**,** 208.

ZAREBSKA, A., AHMETOV, I. I., SAWCZYN, S., WEINER, A. S., KACZMARCZYK, M., FICEK, K., MACIEJEWSKA-KARLOWSKA, A., SAWCZUK, M., LEONSKA-DUNIEC, A. & KLOCEK, T. 2014. Association of the MTHFR 1298A> C (rs1801131) polymorphism with speed and strength sports in Russian and Polish athletes. *Journal of sports sciences,* 32**,** 375-382.

ZEMPO, H., TANABE, K., MURAKAMI, H., IEMITSU, M., MAEDA, S. & KUNO, S. 2010. ACTN3 polymorphism affects thigh muscle area. *International journal of sports medicine,* 31**,** 138-142.

ZMIJEWSKI, P., CIESZCZYK, P. & AHMETOV, II 2018. The NOS3 G894T (rs1799983) and-786T/C (rs2070744) polymorphisms are associated with elite swimmer status. *Biol Sport,* 35**,** 313-319.

Table S3. Polymorphisms used in genotyping, identification of allele-specific probes, and nucleotide reporting based on forward and reverse strand

| SNPs | VIC® | FAM® | Forward strand  (5’-3’) | Reverse strand  (5’-3’) | |
| --- | --- | --- | --- | --- | --- |
| *ACTN3* rs1815739 | T-allele | C-allele | CTGAC[T/C]GAGAG |  | |
| *ACE* rs4341 | C-allele | G-allele | TCAAG[C/G]CATTC |  | |
| *CNTF* rs1800169 | A-allele | G-allele | TCCTC[A/G]GCCAG |  |  |
| *MSTN* rs1805086 | C-allele | T-allele | GGGCC[C/T]TTACT |  | |
| *COL1A1* rs1800012 | A-allele | C-allele | CGCCC[A/C]CATTC |  | |
| *VDR* rs2228570 | A-allele | G-allele | CCTCC[A/G]TCCCT |  | |
| *TRHR* rs7832552 | C-allele | T-allele | AGGTA[C/T]GAATG |  | |
| *PTK2* rs7843014 | A-allele | C-allele | ACCTA[A/C]GAAGT |  | |
| *PTK2* rs7460 | A-allele | T-allele |  | CAATG[T/A]TATTT | |
| *IGF1* rs35767 | A-allele | G-allele | TTTCC[A/G]CATGA |  | |
| *IL6* rs1800795 | C-allele | G-allele | CTTGC[C/G]ATGCT |  | |
| *ACVR1B* rs2854464 | A-allele | G-allele | CAGCC[A/G]TGGGA |  | |
| *ACVR1B* rs10783485 | G-allele | T-allele | CCGCT[G/T]TGATA |  | |
| *ESR1* rs1999805 | A-allele | G-allele | ATTGT[A/G]AATGC |  | |
| *ESR1* rs4870044 | C-allele | T-allele | GGAGA[C/T]GTTGA |  | |
| *MTHFR* rs1537516 | A-allele | G-allele | TCCTA[A/G]GCGGG |  | |
| *MTHFR* rs17421511 | A-allele | G-allele | CTGGA[A/G]TCTGA |  | |
| *MTHFR* rs1801131 | G-allele | T-allele | CACTT[G/T]CTTCA |  | |
| *HIF1A* rs11549465 | C-allele | T-allele | TGTCA[C/T]CATTA |  | |
| *ID3* rs11574 | C-allele | T-allele | CGGAG[C/T]GAGCT |  | |
| *NOS3* rs1799983 | G-allele | T-allele | GATGA[G/T]CCCCC |  | |
| *FTO* rs9939609 | A-allele | T-allele | AATTT[A/T]GTGAT |  | |
| *CNTFR* rs2070802 | A-allele | T-allele | TAATC[A/T]CTGGC |  | |
| *TTN* rs10497520 | C-allele | T-allele | AACTT[C/T]AGGTT |  | |
